# Associations Among Minority Stress, Allostatic Load, and Drug and Alcohol Use in Sexual Minorities: Protocol for the Queer Health Study—a Longitudinal Feasibility Evaluation

**DOI:** 10.2196/73070

**Published:** 2025-06-27

**Authors:** Nathan Grant Smith, Tzuan A Chen, Robert-Paul Juster, Ezemenari M Obasi, Jacob S Crocker

**Affiliations:** 1 Department of Psychological, Health, and Learning Sciences College of Education University of Houston Houston, TX United States; 2 Department of Psychiatry & Addiction Université de Montréal Montreal, QC Canada; 3 Research Center of the Montreal Mental Health University Institute Montreal, QC Canada; 4 Center on Sex*Gender, Allostasis, & Resilience (CESAR) Montreal, QC Canada; 5 Division of Research & Innovation Wayne State University Detroit, MI United States

**Keywords:** sexual and gender minorities, allostatic load, substance use, minority stress, biomarkers, LGBTQ+, health disparity

## Abstract

**Background:**

Substance use rates among sexual minorities are disproportionately greater than that of their heterosexual counterparts. Minority stress theory posits that one explanation for disproportionate substance use in sexual minority populations is a result of increased social stress associated with holding a minoritized identity. This minority stress has been linked to a myriad of negative mental health outcomes, including alcohol and drug use. In addition, emerging research has begun to demonstrate links between minority stress and stress physiology dysregulation. While animal and human models have demonstrated links between stress physiology dysregulation and substance use outcomes, to date, no studies have examined the role that stress physiology plays within a minority stress framework in predicting substance use among sexual minorities. The Queer Health Study was designed to explore the longitudinal links among minority stress, stress physiology (specifically, allostatic load, the cumulative “wear and tear” on the body and brain as a result of chronic stress), and substance use.

**Objective:**

This study aims to assess the feasibility of collecting longitudinal data to explore the temporal links between minority stress processes, allostatic load, and drug and alcohol use, as well as to obtain estimates of effect size to determine the appropriate sample size necessary to conduct a fully powered longitudinal study.

**Methods:**

This feasibility study is a 3-wave longitudinal design consisting of a self-report survey, researcher-assisted Timeline Followback to assess for drug and alcohol use, and blood and anthropometric data collection to measure allostatic load at each of the time points. A total of 40 ethnically and racially diverse sexual minority adult participants (aged 18-60 years) will be enrolled.

**Results:**

The study received University of Houston institutional review board approval on July 31, 2023 (STUDY00004277). Recruitment began in June 2024. As of February 2025, the initial sample of 46 participants completed the time 1 visit, and time 2 visits are ongoing. We estimate that all study activities will be completed by July 2025.

**Conclusions:**

Results can inform the development of targeted prevention and treatment interventions. In addition, this research will provide an innovative framework for exploring diverse risk and resilience factors impacting addiction in this at-risk population. Ultimately, results have important implications for public health and have the potential to reduce the many dire economic and health consequences of drug use and addiction.

**International Registered Report Identifier (IRRID):**

DERR1-10.2196/73070

## Introduction

### Background

Sexual minority (SM) adults disproportionally use drugs and are at greater risk for substance use disorders (SUDs) than their heterosexual counterparts. National data revealed that SM adults were more than twice as likely as heterosexuals (39.1% vs 17.1%) to have used drugs in the past year [1]. Likewise, SM women (60.8% vs 24.3%) and SM men (65% vs 49.9%) are more likely to have a lifetime SUD compared to their heterosexual counterparts [2]. Drug use is estimated to cost society over US $193 billion annually [3,4]. Given the documented disparity in drug use and SUDs among SM adults, it is critical to understand the factors that mediate risk and resilience for drug use and abuse.

One explanation for high rates of drug use and SUDs among SMs is that increased stress related to being an SM confers risk for drug use. This social stress has been termed minority stress [5,6] and has been linked, primarily in cross-sectional research, to a variety of negative mental health outcomes [7] and alcohol and drug use [8,9].

Nascent research has begun to demonstrate that minority stress is related to stress physiology dysregulation [10]. This research demonstrated that SMs who had disclosed their sexual orientation had lower concentrations of the stress hormone cortisol than those who had not disclosed [10]. Related, Doyle and Molix [11] found that gay men who experienced more discrimination had higher levels of the inflammatory cytokine interleukin (IL)-6. Another study found that SM young adults who were raised in highly stigmatizing environments demonstrated a blunted cortisol response to a social stressor [12].

In addition to limited studies linking minority stress processes to individual markers of stress physiology, research has revealed that SMs, compared to heterosexuals, demonstrate different stress physiology patterns. For example, Juster et al [13] showed that SM men had a lower allostatic load (AL) than heterosexual men. Also, SM women displayed higher cortisol reactivity than heterosexual women; SM men, on the other hand, showed lower cortisol reactivity than heterosexual men. Mays et al [14] found that bisexual men had significantly higher AL than heterosexual men but gay men had lower AL than heterosexual men.

Currently, only a handful of studies exist that examine minority stress constructs in the context of stress physiology. However, no studies to date have examined the link between stress physiology and drug use in SM samples. Research in other populations suggests that stress physiology dysregulation, specifically, the concept of AL, may be a promising avenue for understanding drug use vulnerability and trajectory.

AL refers to the cumulative “wear and tear” on the body and brain due to repeated activation of allostatic responses resulting from chronic stress [15]. Prolonged secretion of stress hormones, through the hypothalamic-pituitary-adrenal (HPA) axis and sympathetic-adrenal-medullary axis, results in strain on interdependent systems and subsequent physiological dysregulation. Moreover, AL confers risk for accelerated aging, worsened disease trajectories, and all-cause mortality [16]. In both animal and human research, chronic stress, subsequent HPA axis dysregulation, and allostatic states, including AL, are linked to both the initiation of and trajectories of drug use [17] and addiction [18]. Indeed, stress and addiction neurocircuitry are intertwined [19].

At a theoretical level, drug addiction fits within an allostatic model in that it challenges the brain circuits involved in emotional regulation to exacerbate addiction cycles of binge and intoxication, withdrawal and negative affect, and preoccupation and anticipation [18]. Under conditions of chronic drug abuse and relapse, reward and stress pathways become dysregulated and contribute to allostatic states and AL via the HPA axis and downstream effects [20]. For example, Wand et al [21] found that stress-induced cortisol was positively correlated with both amphetamine-induced mesolimbic dopamine and the subjective positive effects of amphetamine, both risk factors for drug use initiation and addiction. Moreover, AL has been implicated in the transition from drug use to addiction, and the AL framework helps to explain the biological mechanisms undergirding both initial use and subsequent addiction [22,23].

No published research to date has explored the link between AL and drug use in SM adults. Given that SM adults face unique, chronic stressors, which are linked to AL, and are more likely than their heterosexual counterparts to use drugs and be diagnosed with SUDs, there is an urgent need to explore the mechanisms underlying drug use outcomes in this at-risk population. In addition, limited research has measured AL repeatedly over time (in any population) to explore the link between AL change and subsequent health outcomes. The limited data suggest that AL variability over time may be an important predictor of health [24]. Thus, more research is needed to understand the longitudinal links between changes in AL and health outcomes.

### The Current Research

**Figure 1:**
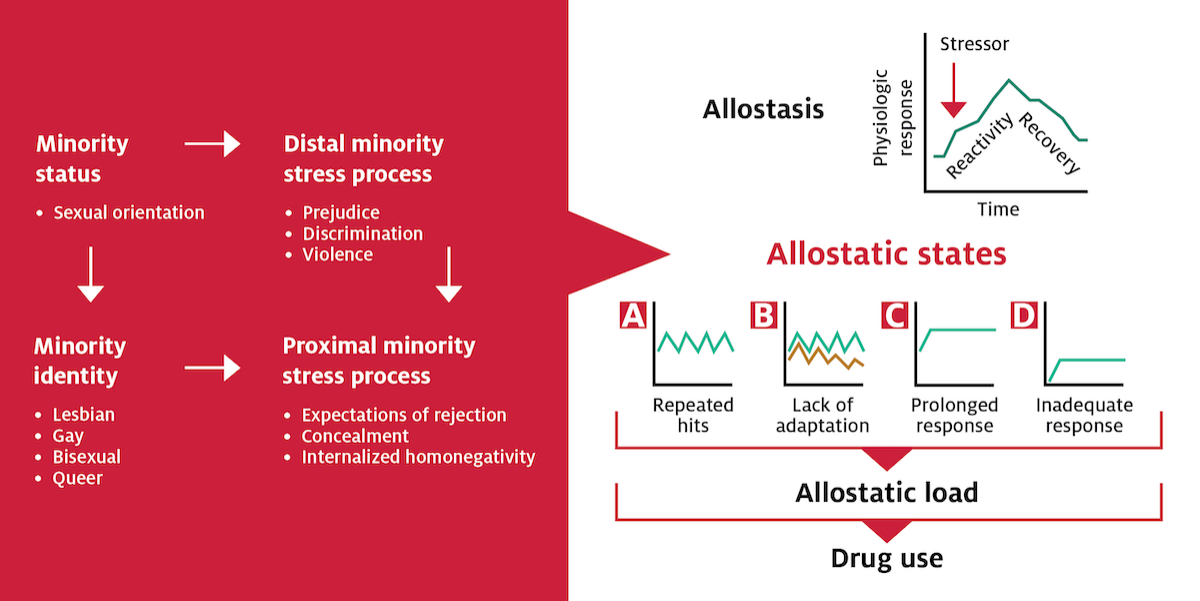
Conceptual model.

To date, no studies have comprehensively measured minority stress (ie, discrimination, internalized homonegativity, concealment, and expected rejection [5]) in research examining SM biomarkers. Moreover, most research examines single markers of stress physiology. Next, the majority of research is cross-sectional. Thus, there is a need to fully explore the impact of minority stressors on a comprehensive measure of stress physiology dysregulation using longitudinal methods. Finally, no research exists to examine the links between stress physiology and drug use in SM adults, a population with demonstrated drug use and SUD disparities. Thus, this study proposes that minority stressors resulting from minority sexual orientation lead to allostatic states and increased AL, which, in turn, confers risk for drug use. Figure 1 (adapted from Juster et al [25]) provides an overview of our conceptual model.

### Objective

This study aims to examine the feasibility of collecting longitudinal data to examine the relationships between minority stress, AL, and drug use, following a sample of SM adults over the course of 1 year. This innovative study will be the first to explore the links between AL and drug use in this population, which has demonstrated drug-use health disparities.

This protocol proposes 3 aims:

1. Assess the feasibility of collecting longitudinal data to explore the links among minority stress processes, AL, and drug use. It is hypothesized that participants will find the study procedures acceptable, there will be minimal attrition over time, there will be minimal missing data, and the target sample size will be successfully recruited.
2. Obtain estimates of effect size. Pilot data will be used to obtain estimates of effect sizes between minority stress and AL, AL and drug use, and minority stress and drug use over 3 repeated-measures time points. These effect sizes will be used to determine the necessary sample size to conduct a fully-powered longitudinal study over a longer time period (eg, 6 time points over 2 years).
3. Explore the temporal links between minority stress, AL, and drug use. Because physiological dysregulation has been linked to the initiation of drug use, and drug use has been linked to subsequent physiological dysregulation, an exploratory aim (given the limited sample size) of this research is to test 2 competing models: (1) minority stress→AL→drug use versus and (2) minority stress→drug use→AL.

## Methods

### Ethical Considerations

The study received University of Houston institutional review board approval on July 31, 2023 (IRB ID: STUDY00004277).

### Background

In order to test the proof of concept and feasibility, we will conduct a prospective longitudinal design with 3 time points over the course of 1 year (ie, every 4 months). These 3 time points were chosen to reduce participant burden while maximizing our ability to examine change over time. Moreover, prior research has shown statistically significant changes in AL over a 4-month period, with those who abstained from alcohol being more likely to evidence decreases in AL over 4 months [26].

A total of 40 ethnically and racially diverse SM adult participants (aged 18-60 years) will be targeted. A sample of 40 participants is consistent with sample size recommendations for pilot feasibility studies [27,28]. To address potential attrition, the first time point will over enroll by approximately 20% (approximately 48 participants to be enrolled at time 1). Participants will be limited to the age range of 18-60 years because of the impact of natural aging on AL [16]. This age range was chosen because AL steadily increases from the age of 25 years to 60 years and then plateaus [29]. Limiting to adults aged <61 years will allow us to target the age group that shows the greatest shifts in AL, maximizing chances of detecting meaningful change.

We expect to have equal proportions of female-assigned-at-birth and male-assigned-at-birth participants. Transgender individuals who also identify as SMs will be included. The racial and ethnic makeup of the sample is expected to mirror that of Houston, which includes non-Hispanic White (24.4%), Black or African American (22.6%), Asian or Asian American (6.8%), and Latin or Hispanic individuals (45.0%) [30].

Participants will be recruited in the metropolitan Houston, Texas, area through advertisements and events in community venues. Advertisements will include flyers in SM-inclusive bars, restaurants, and retail shops; in print/electronic advertisements (eg, Facebook, Instagram, and Grindr); and at SM community events, such as the annual Houston pride celebration (700,000 attendees annually).

Inclusion and exclusion criteria include (1) self-identification as lesbian, gay, bisexual, queer, or other nonheterosexual identity; (2) age of 18-60 years; (3) willingness to complete study tasks; (4) valid email address (for maintaining contact, such as sending appointment reminders); and (5) proficiency in English. Failure to meet any inclusion criterion will result in study exclusion.

### Design

This study involves a prospective longitudinal design with 3 time points over the course of 1 year (ie, every 4 months). Participants come to the laboratory every 4 months over the course of 1 year to provide biomarkers and self-report data. At each visit, participants will complete computer-administered (Qualtrics) self-report measures and a researcher-assisted Timeline Followback to assess for drug and alcohol use. Participants will provide blood samples to assess biomarkers and have anthropometric data collected to assess for adiposity and blood pressure.

Participants will be compensated in retail gift cards (ie, Tango) for the completion of specific portions of the study. They will receive US $50 for completing each of 3 in-person assessment visits (US $150 total) and an additional US $50 for the completion of all 3 in-person assessments. In total, a participant can receive up to US $200 in retail gift cards for participating in the study.

Interested participants will complete an initial phone screening to assess for inclusion criteria. Those meeting all inclusion criteria will be given a scheduled time to come to the laboratory. Eligible participants will then complete blood draw, anthropometric measurements, and self-report measures.

### Measures

At each of 3 time points, participants will complete self-report questionnaires (via Qualtrics), containing a demographic questionnaire and the measures below. All measures have good reliability and validity, and have been used with SM samples. Demographics will assess sexual orientation, sex assigned at birth, current gender identity, race and ethnicity, age, income, education, employment, occupation, relationship status, and current medications. Internalized homonegativity will be measured using the Internalized Homophobia Scale of Theodore et al [31] with edits to be inclusive of all genders, per Edwards et al [32]. Discrimination will be measured using Szymanski’s [33] Heterosexist Harassment, Rejection, and Discrimination Scale. Anticipated discrimination will be measured using the Gay-Related Rejection Sensitivity Scale of Pachankis et al [34] with edits to be inclusive of all genders, per Feinstein et al [35]. Sexual orientation concealment will be measured using Jackson and Mohr’s [36] Sexual Orientation Concealment Scale [36]. Intersectional stress will be measured by the Everyday Identity Stress Scale of Mereish et al [37].

Participant acceptability will be measured using quantitative and qualitative questions from the Theoretical Framework of Acceptability [38,39].

Study feasibility will be assessed via participant recruitment, enrollment, and attrition, which will be tracked following CONSORT (Consolidated Standards of Reporting Trials) guidelines [40].

In addition, we will track how long it takes to recruit the sample and calculate the proportion of missing data, usable biomarker data, participants eligible after screening, participants consented, and participants retained at each time point.

Drug and alcohol abuse consequences will be assessed via the Drug Abuse Screening Test (DAST-10) [41] and the Alcohol Use Disorders Identification Test (AUDIT) [42]. Depression will be measured by the Center for Epidemiological Studies Depression Scale [43]. Anxiety will be measured by the Generalized Anxiety Disorder-7 Scale [44]. Sleep quality will be measured using the Pittsburgh Sleep Quality Index [45]. Belongingness will be measured using the General Belongingness Scale [46]. Drug and alcohol use frequency will be assessed using 120-day Timeline Followback [47] for the following substances: alcohol, cannabis, cocaine, phencyclidine, opiates, methamphetamine (including 3,4-methylenedioxymethamphetamine and ecstasy), hallucinogens, methadone, heroin, amphetamines, barbiturates, benzodiazepines, inhalants, and synthetic cannabinoids.

Smoking will be measured using standard questions from the National Health Interview Survey [48]. Vaping will be measured by the Penn State Electronic Cigarette Dependence Index [49].

#### Physiologic and Anthropometric Measures

Biomarkers will include immune functioning indicators (IL-1β, -6, and -8, tumor necrosis factor-alpha, C-reactive protein, serum amyloid A, vascular cell adhesion molecule 1, and intercellular cell adhesion molecule 1), metabolic functioning indicators (glycosylated hemoglobin, high-density lipoprotein, and low-density lipoprotein cholesterol, and triglycerides), adiposity (BMI, body fat percent, and waist-to-hip ratio), and blood pressure.

#### Blood Collection and Assaying

A total of 10 mL of blood will be collected via venipuncture at the University of Houston by a certified phlebotomist. Samples will be centrifuged, and plasma will be extracted and aliquoted into cryovials for storage at –80 °C until assaying. All samples will be tested in duplicate; samples from the same participant will be assayed on the same plate within the same run. Validated V-PLEX Viral Panel 1 kits (ie, IL-1β, IL-6, IL-8, and tumor necrosis factor-α) and Vascular Injury Panel 2 kits (ie, C-reactive protein, serum amyloid A, vascular cell adhesion molecule 1, and intercellular cell adhesion molecule 1) from Meso Scale Discovery will be used to assay these biomarkers using the MESO QuickPlex SQ 120. Glycosylated hemoglobin will be measured using a Siemens DCA Vantage Analyzer. High-density lipoprotein and low-density lipoprotein cholesterol and triglycerides will be measured using CardioChek Plus, a standard point-of-care device.

#### Adiposity

Weight, percent body fat, muscle mass, bone mass, and percent body water will be measured using a Tanita DC-430U dual-frequency total body composition analyzer. Height will be measured using a Seca (model 217) stadiometer. BMI will be calculated using the standard formula. Body fat percentage will be calculated using sex-specific formulas (for trans participants, the average of the male and female ranges will be used). The waist-to-hip ratio will be measured with an ergonomic Seca (model 201) measuring tape, measured in cm, dividing the waist by hip circumference.

#### Blood Pressure

Blood pressure will be measured using an electronic sphygmomanometer (Omron model BP785N). The average of 3 systolic and 3 diastolic resting readings, taken while sitting, will be computed.

#### Allostatic Load

AL will be calculated using an established count-based approach [10,50,51]: for each measure, a clinical cutoff will be used such that those falling above the cutoff will score 1, while those falling below will score 0; scores will be summed to calculate AL index. This approach to AL index calculation has been used in previous studies of SM adults [10]. As an alternative to the most commonly used count-based AL index, we also will calculate a *z* score AL index that represents the sum of an individual’s obtained *z* scores for each biomarker based on the sample’s distribution of biomarker values, allowing the relative weight of each biomarker to be different depending on its deviation from the sample’s mean [16]. We will annotate the sample collection date and time so that time can be controlled for analyses. Likewise, we will assess for current medications at each time point and control in analyses.

### Participant Retention Plan

Because the research aims to follow participants over three time points, we will implement several efforts to reduce attrition [52,53]. First, regular communication from the research team will be sent to participants (with opt-in consent), such as health information communication, to continue to engage participants. We will use a graphics designer to develop a “project identity” [54] so that all paper and electronic materials have a consistent look and feel, which has been shown to increase participant identification with research projects and minimize the possibility that communication will be discarded or overlooked. Next, participant tracking procedures will be followed and will include sending reminders of upcoming appointments using participants’ preferred contact methods (phone call, social media direct messaging, or email), and following up with participants who miss appointments. We will offer appointments both during the day and evenings, to help accommodate participant work schedules. Next, participants who complete all 3 assessments will be given an additional US $50 gift card. Finally, participant tracking will be integrated with research staff training; as well, participant tracking strategies and success or lack thereof will be regularly evaluated and addressed in research team meetings.

### Data Analytical Plan

To address aim 1, we will track how long it takes to recruit the sample and calculate the proportion of missing data, useable biomarker data, participants eligible after screening, participants consented, and participants retained at each time point. Measures of central tendency and dispersion will be calculated for acceptability measures. Qualitative acceptability data will be analyzed via content analysis and any recommendations from participants regarding adaptation of study procedures will be evaluated for refinement of future large-scale implementation of the protocol.

To address aim 2 and exploratory aim 3, we will first examine the missing data pattern and apply imputation procedures for the missing responses following dropout, such as multiple imputation, in which each missing value is replaced with several plausible values [55]. Next, field and range checks will be conducted. Distributional characteristics will be assessed and outliers will be checked. Prior to inferential procedures, extensive descriptive statistical analyses of the outcome and predictor variables will be conducted. Standard descriptive statistics including means, SDs, ranges, box plots, histograms, and frequencies will be calculated. Normalizing transformations will be explored as appropriate. For aim 2, estimates of effect size will be calculated via Pearson correlations (or Spearman correlations, depending on distribution).

For exploratory aim 3, a longitudinal path modeling using a fully cross-lagged design [56] will be used to explore whether (1) minority stress impacts AL, which in turn impacts drug use or (2) minority stress impacts drug use, which in turn impacts AL. Because minority stress and AL will be measured at the same time, the casual directions between them cannot be established. Therefore, we will use a cross-lagged design, a special case of structural equation modeling [57], to estimate autoregressive and cross-lagged paths, allowing us to address the reciprocal influences of minority stress and AL simultaneously. Because of the longitudinal model, the autoregressive effects are modeled as lags where baseline measures are predictors of post-1 measures and post-1 measures are predictors of post-2 measures; thus, the temporal order is preserved. Given the small sample size and exploratory nature of aim 3, the model will be underpowered and at risk for increased likelihood of type II errors. As such, the results of inferential statistics will need to be interpreted with caution. Nonetheless, we will be able to observe trends and calculate descriptive statistics in the exploratory data. All analyses will be conducted using SAS (version 9.4) [58] and Mplus (version 8.4) [59].

## Results

Funding for the study was approved by the National Institute of Minority Health and Health Disparities on August 1, 2023. Recruitment began in June 2024. As of February 2025, an initial sample of 46 participants completed the time 1 visit. Time 1 was oversampled to account for anticipated attrition with the goal of achieving a total sample size of 40 that completes all time points. Approximately half of the initial sample has completed the time 2 visit, and assessments are ongoing. We estimate that all study activities will be completed by July 2025.

## Discussion

It is expected that the protocol will be feasible with high participant satisfaction. The results will also provide estimates of effect size, which we will use to develop a fully powered study to be conducted over a longer time period (eg, 6 visits over 2 years). With a fully powered and larger sample size, we will be able to minimize the likelihood that the model falsely leads to a type II error. More importantly, the results will provide novel insights into the mechanisms and trajectories of drug addiction in an at-risk population and provide a useful framework for examining the role of stress physiology dysregulation in other populations at-risk for drug use disparities.

This innovative protocol is the first to propose a longitudinal study of sexual minority biopsychosocial determinants of substance use. As the only research to date that explores the interplay of neurobiological and psychosocial risk factors in drug use outcomes among SM adults, a population with demonstrated disparities in drug use and SUDs, the current research will provide an innovative framework for future research to explore diverse risk and resilience factors impacting addiction in this at-risk population. As such, this research addresses a critical health disparity.

Moreover, the outcomes of this research will provide important insights into the potential mechanisms of action for substance use trajectory and addiction, as well as offer implications for the development of interventions to reduce and prevent substance use. If results support the longitudinal links between minority stress and substance use through stress physiology dysregulation, researchers will be provided with novel insights into the mechanisms driving substance use in this population.

In addition, such results will provide clear direction for intervention development. For example, the results can influence the development of tailored interventions to directly target AL among SMs. Indeed, reducing AL has been demonstrated to be beneficial. Research in older adults has revealed that reductions over time in AL are protective against all-cause mortality, with a mortality rate of 15% for those whose AL increased over 2.5 years but only 5% for those whose AL decreased over the same time period [24]. In addition, research has revealed that behavioral interventions can reduce AL. For example, stress-reduction interventions such as transcendental meditation and yoga have demonstrated effects on reductions in AL-related variables such as blood pressure [60] and cortisol [61]. Interventions targeting minority stress in SM adults also have been developed and show promise for addressing substance use problems (eg, [62,63]). Thus, interventions that target both minority stress factors and AL may have the potential to move the needle on SM substance use outcomes. Results from this study will offer unique insights that can be used to develop tailored, interdisciplinary interventions that target multiple, modifiable risk factors for addiction.

Next, the methodological framework developed and tested in the research has the potential to serve as a model for exploring factors influencing addiction in other underrepresented populations with health disparities (see, for example, research on links between physiological dysregulation and drug use in African American young adults [64]). As noted, the results can also be used to develop innovative prevention, early detection, and treatment efforts to reduce drug use and addiction (in this and other populations) based on understanding how the interactions of biological and psychosocial factors influence drug use trajectories.

There are some limitations of the protocol to note. Participants are required to complete multiple assessments, including psychological, behavioral, and physiological measures over multiple time points. The battery has the potential to increase participant burden and impact dropout. We will review responses to our qualitative acceptability questions to determine if participants noted fatigue or viewed the protocol as burdensome and use such data to refine future studies. We will also explore attrition rates. Of note, we previously enrolled SM adults in Houston, Texas, in another study that included in-person data collection with lengthy surveys and multiple anthropometric and physiological measures. Though we did not assess for acceptability, we did not receive any unsolicited feedback regarding participant fatigue and we had minimal missing data. Moreover, the study aims were found to be feasible [65]. As noted previously, the small sample size runs the risk of type II errors, so quantitative results will be interpreted cautiously. Despite these limitations, this innovative study will provide valuable feasibility and acceptability data that will form the basis of a subsequent fully-powered study and will offer unique insights into drivers of SM substance use.

Ultimately, the research described in this protocol has the potential to reduce the many dire economic and health consequences of drug use and addiction. Moreover, the research represents a critical step in eliminating health disparities that reduce quantity and quality of life and increase health care costs.

## Abbreviations

AL: allostatic load
AUDIT: Alcohol Use Disorders Identification Test
CONSORT: Consolidated Standards of Reporting Trials
DAST-10: Drug Abuse Screening Test
HPA: hypothalamic-pituitary-adrenal
IL: interleukin
SM: sexual minority
SUD: substance use disorder
